# Olive Pomace Oil Structuring for the Development of Healthy Puff Pastry Laminating Fats: The Effect of Chilling Storage on the Quality of Baked Products

**DOI:** 10.3390/foods13040603

**Published:** 2024-02-16

**Authors:** María Dolores Álvarez, Arancha Saiz, Beatriz Herranz, Susana Cofrades

**Affiliations:** 1 Institute of Food Science, Technology and Nutrition (ICTAN-CSIC), c/José Antonio Novais, 6, 28040 Madrid, Spain; a.saiz@ictan.csic.es (A.S.); scofrades@ictan.csic.es (S.C.); 2 Department of Food Technology, Veterinary Faculty, Complutense University, Avda/Puerta de Hierro, s/n, 28040 Madrid, Spain

**Keywords:** structured olive pomace oil, puff pastry laminating fat, physicochemical properties, chilling storage, static crystallization, graininess, baking performance

## Abstract

Developing puff pastry (PP) laminating fats (LFs) with sustainable structured olive pomace oil (OPO) could contribute to its increased valorization. This study evaluated the physicochemical stability of four OPO-based LFs or margarines and the performance of their baked PP counterparts during two months of chilling storage at 4 °C. LF samples, developed at the laboratory scale, contained 41% (LF1 and LF2) OPO and 31% (LF3 and LF4) OPO together with 10% cocoa butter when using two static initial crystallization conditions (room temperature for LF1 and LF3, freezer for LF2 and LF4) before storage. During the storage period, the proximate composition, thermal and dynamic rheological properties, firmness and spreadability, oil-binding capacity, color, and lipid oxidation of the four LF samples were examined, along with the baking performance and textural properties of the PP counterparts. The initial cooling rate had minimal significance. Cocoa butter negatively influenced post-crystallization processes occurring in OPO-based LF3 and LF4, resulting in increased hardness and reduced performance after 18 days of storage, attributed, at least partially, to a high amount of 1,3-dipalmitoyl-2-oleoyl-glycerol (POP), mainly from cocoa butter. Conversely, OPO-based LF1 and LF2 maintained their quality and were stable for two months without apparent granular crystal formation.

## 1. Introduction

Puff pastry (PP), particularly valued for its light and flaky texture, is a baked laminated dough or paste prepared from a mixture of basic dough and margarine or an LF. This type of PP is leavened without the use of yeast or any rising agents [1,2]. 

Butter is generally used as the LF in PP products due to its appreciated flavor associated with its milk fat composition. Milk fat has approximately 400 types of fatty acids (FAs) with chain lengths varying from C4 to C18 and an extensive range of molecular weight species of triacylglycerols (TGAs) (from 26 to 56 carbon atoms) [3]. Consequently, milk fat melts within a broad temperature spectrum (−40 to 40 °C) [4]. TAG composition is one of the most important chemical characteristics that determine the physical properties of fats and oils, playing an essential role in the crystallization process [3,4]. However, the composition of butter milk fat presents the undesirable disadvantage of having ~65% saturated fat, and butter also presents the drawback of having low spreadability at refrigeration temperatures (4–10 °C) [3,4]. As a result, it does not spread immediately when taken out of the refrigerator [5], hindering paste folding and sheeting operations during PP production.

Palm oil-based LF, free from hydrogenated and trans fats [6], is a viable alternative to butter and a highly valuable hard fat within the lipid industry [7,8]. Palm oil has the distinctive property of having an equal proportion of saturated fatty acids (SFAs) and unsaturated fatty acids (UFAs), with about 39% monounsaturated, about 10.5% polyunsaturated, and around 50% saturated FAs [6]. Given its FA composition, palm oil tends to form small beta prime (β′) polymorph crystals, which are highly stable during long-term storage. The major symmetric disaturated TGA of palm oil is POP, while it also contains a significant amount of asymmetric diunsaturated 1-palmitoyl-2,3-dioleoyl-glycerol (POO) [6,9]. On the other hand, palm oil also exhibits high oxidative stability, attributed to its high content of antioxidants, such as tocopherols and tocotrienols, and its low concentration of the aforementioned polyunsaturated FA (PUFA).

Nevertheless, in the formulation of palm oil-based LF, numerous studies have shown that palm oil is frequently combined with other oils or with its common fractions (palm stearin (PS) and palm olein) [7,9] or with milk fat [10,11]. PS is high in C48 tripalmitin (PPP), with a high melting point ranging from 44 to 56 °C [9]. Hardstock PS provides structure to the products, being particularly desirable in the formulation of margarines, in which the beta (β) polymorph is required to provide flakiness [6].

In Spain, in recent years, much effort has been focused on the valorization of olive pomace oil (OPO), as this country is the world’s leading producer of this oil [12]. Crude OPO is obtained from the wet olive paste, or alperujo, a solid by-product consisting of the remains of olive skin, pulp, and stones, which is obtained during the mechanical extraction process of virgin olive oil [12,13,14,15,16,17]. In turn, from the wet olive paste, OPO extraction with *n*-hexane and refining processes generate more substantial quantities of by-products. However, the pomace sector efficiently utilizes 100% of these by-products, generating compost and biomass for energy applications. This approach provides important environmental benefits, as well as antioxidants (phenols and hydroxytyrosol) with nutritional applications. Therefore, the consumption of refined OPO in Spain fosters the sustainability of the olive oil chain, promotes a circular economy, and minimizes waste by producing zero residues.

On the other hand, OPO is also one of the four categories of olive oils recognized in European legislation [18], all of which present similar lipid composition [12]. Consequently, in refined OPO oil, oleic acid (C18:1n9) is the major FA (~73%), followed by palmitic (C16:0) (~11%) and linoleic (C18:2n6) (~10%) acids; therefore, it has much lower SFA (~16%) and higher UFA contents (~85%) than palm oil [15,16,19,20]. As expected, OPO possesses a suitable TAG composition, mainly composed of triunsaturated triolein (OOO), followed by POO and polyunsaturated 1-linoleoyl-2,3-dioleoyl-glycerols (LOO). Regardless of its lipid composition, OPO contains other recognized unique minor components with biological activities, such as triterpenic compounds and aliphatic alcohols, which determine its health-promoting potential, particularly for cardiovascular health [12,13,14,21].

In addition to its beneficial properties, OPO has proven to be very suitable for frying processes due to its high resistance to oxidative degradation at high temperatures. This is attributed to its high monounsaturated-to-polyunsaturated FA ratio, along with the added positive effect of β-sitosterol and squalene as minor compounds [15,22]. Also, due to its unique oleic-to-linoleic FA ratio, OPO is highly suitable for bakery products, with higher resistance to oxidative changes than sunflower oils during the processing and shelf-life of cupcakes. In addition, the bioactive components of OPO remained practically unchanged after cupcakes were baked and stored at room temperature for 6 months. Moreover, Alvarez et al. [16,23] recently demonstrated that OPO shows technological applicability to the formulation of margarines. Therefore, previous studies [16,17] provide evidence that OPO could be a healthier alternative to palm and sunflower oils in the confectionery industry, although further research is still required.

In fact, for a TAG to exist in the β′ crystal form, its carbon number should be kept as low as possible, preferably below C54, and OPO is rich in TGAs with carbon number 42 [24]. However, the TAG composition also significantly determines the post-crystallization processes that occur mainly in palm oil-based margarines and shortenings [6,7,8]. Post-crystallization is a common hardening phenomenon that takes place after production and is related to the processing of non-hydrogenated LFs [6]. This phenomenon increases the hardness of finished products several weeks after production, and it could result from either the transformation of the β′ to β form of crystals or the growth of β crystals during storage and is detrimental to margarine quality [8,10,11]. Post-crystallization is also related to the crystallization rate, and formulations containing high amounts of palm oil tend to crystallize slowly due to their high content of low-melting TAGs [6]. In LFs, the amounts of POP, PPO, and PPP should be carefully equilibrated to avoid unwanted crystal network rearrangements, which could lead to increased hardness and a significant reduction in baking performance [7].

In a previous study focused on formulations with OPO [16], four LF samples containing 41% OPO and 31% OPO combined with 10% cocoa butter were developed for the first time in the laboratory under two static crystallization conditions. All of them resulted in PP with very acceptable baking performance, excellent sensory quality, and a healthy lipid profile. As a continuation of that study, the aim of the present work was to evaluate the chilling storage stability and functionality of these four OPO-based LFs or margarines over a period of two months, because it is also important to ensure the long-term stability of the formulated OPO-based LFs. For this purpose, the thermal properties, rheological and textural properties of the fat crystal network, color, oil-binding capacity, and lipid oxidation were measured during the course of storage, along with baking performance. The behavior of the four LFs and their PP counterparts was compared with that of the following controls: a commercial butter (CB) and LF (CLF) and baked products prepared with the CB and CLF (PPCB and PPCLF).

## 2. Materials and Methods

### 2.1. Materials

The following ingredients were part of the LF oil phase (OP): OPO, refined palm stearin (PS) flakes, salted table butter, cocoa butter, beeswax, and emulsifiers: Verolec non-GMO IP prepared with soybean lecithin (E-322); Verol N-90, distilled monoglycerides (E-471); Verol P, polyglycerol ester of FA (E-475) were all provided by Lasenor (Barcelona, Spain); and Palsgaard^®^ 1311, consisting of a blend of polyglycerol esters of FAs and citric esters of monoglycerides and diglycerides of vegetable FAs, was provided by Palsgaard A/S (Juelsminde, Denmark). The refined OPO used in the study was produced in Spain, supplied by Interprofesional del Aceite de Orujo de Oliva (ORIVA, Sevilla, Spain), and refined by ACESUR SA (Sevilla, Spain). The LF aqueous phase (AP) was composed of tap water, fine table salt, anhydrous citric acid, ground dietary gelatin 200/220 Bloom, butter flavor, and tartaric acid. The composition, manufacturers, and suppliers of the various ingredients constituting both the LF OP and AP have been previously described in detail [16]. In addition, for the preparation of the basic PP dough, strong flour (Tradicional Zamorana, Zamora, Spain) and wheat flour (Gallo, Córdoba, Spain) were purchased from local retailers. All ingredients and fatty additives were kept under appropriate conditions to protect them from oxidative stress.

### 2.2. Laminating Fat (LF)-Making Procedure and Chilling Storage

LF1, LF2, LF3, and LF4 (LF1–LF4) with an OP/AP proportion of 80/20 were prepared in batches of 1300 g. For the four LF samples developed, the percentage of each ingredient added to both the OP and AP is shown in Table S1. In the OP, the amount of each ingredient was fixed, except for the percentage of OPO, which was 41% in LF1 and LF2 and 31% in LF3 and LF4. In addition, LF3 and LF4 contained 10% cocoa butter. The ingredients and percentages in the AP were the same in the four LFs (Table S1). Both the OP and AP were prepared according to Álvarez et al. [16]. The emulsification process was performed at 50 °C in a Thermomix^®^ TM5-1 homogenizer (Vorwerk, Nordrhein-Westfalen, Germany). The AP (~12 °C) was incorporated into the OP at 1500 rpm for 3 min, followed by an increase in stirring speed up to 10,200 rpm, which was maintained for 30 s. The emulsion was then cooled and stirred at 1500 rpm by placing it in a cold ice bath (~5 °C) for 17 min. The final emulsion temperature was 39 °C. Next, each liquid emulsion was poured into plastic trays containing 330 g of sample, and two initial cooling rates for static fat crystallization were tested. LF1 and LF3 were allowed to rest for 1 h at room temperature (cooling rate = 0.144 °C/min), whereas LF2 and LF4 were kept at −24 °C for 1 h (cooling rate = 0.380 °C/min). In all cases, after the initial crystallization, the plastic trays were covered with an aluminum seal for chilling storage at 4 ± 1 °C for two months. The samples were evaluated after 3, 18, 33, and 60 days of storage. The CB and CLF were not evaluated over time because the thermal history and process conditions of these control samples are likely to be very distinct and unknown to us.

### 2.3. Puff Pastry (PP)-Making Procedure

For each of the four storage times, four baked PPs were prepared with each formulated LF1–LF4 containing OPO (PP1, PP2, PP3, and PP4). They were then compared with two PPs made with a commercial butter (PPCB) and a commercial LF (PPCLF). All PP samples were prepared according to the French method [25,26], where a piece of the corresponding roll-in fat is wrapped with basic dough and folded several times to obtain a multilayered dough or paste. The complete PP-making procedure used was recently described in detail [16]. In each case, six single turns were executed, resulting in 1459 theoretical layers with 729 theoretical fat layers. Square samples of 5 × 5 cm^2^ were cut and arranged in two rows of 6 samples on a tray lined with baking paper. Baking was carried out in a Rational oven (Combi-Master, CM6, Landsberg, Germany) at 220 °C for 12 min for the controls PPCB and PPCLF, while an additional five min at 170 °C was applied for samples PP1–PP4.

### 2.4. Laminating Fat (LF) Proximate Composition

The proximate compositions (moisture, protein, ash content) of the LF samples and both controls CB and CLF were determined after 3 days of chilling storage according to official methods [27]. In turn, fat content was calculated as the difference of the sum of the contents of these three components with respect to 100.

### 2.5. Laminating Fat (LF) Thermal Behavior

A TA Q1000 differential scanning calorimeter (TA Instruments, New Castle, DE, USA) was used to evaluate the thermal characteristics of the LF and compare them to those of the control samples CB and CLF, as well as to those of the major ingredients: OPO, PS, and cocoa butter. The calibration was performed using indium and zinc. Samples of 6–8 mg were weighed and placed into aluminum pans with lids and covered; an empty pan was used as a reference. For cooling thermograms, the samples were equilibrated at 80 °C for 10 min and then cooled from 80 to −10 °C at a constant rate of 5 °C/min. For heating thermograms, the samples were kept at −10 °C for 10 min and then heated from −10 to 80 °C at the same constant rate. Crystallization and melting peak temperatures (T_cp_ and T_mp_, °C) and peak enthalpies (ΔH_cp_ and ΔH_mp_, J/g) were calculated from the cooling and heating thermograms using the DSC Thermal Advantage instruments PC manager software (vs. 1.3 beta) (TA Instruments – Waters LLC, New Castle, DE, USA). Thermal profiles were recorded at the beginning and end (3 and 60 days) of the entire storage chilling period studied.

### 2.6. Laminating Fat (LF) Rheological Measurement

The rheological behavior was evaluated by small-amplitude oscillatory shear (SAOS) tests in a Kinexus pro controlled stress rheometer (Malvern Instruments Ltd., Worcestershire, UK) with a high-temperature cartridge in the lower plate for temperature control. A 20 mm diameter plate–plate geometry with a serrated surface and a 1.5 mm gap was employed. In all cases, a cover cell was used to prevent the sample from drying due to either temperature or time. Before initiating measurements, each sample was given a 5 min resting period to allow the temperature to stabilize. Then, they were subjected to a time sweep test for 20 min at a fixed frequency of 1 Hz and a fixed stress wave amplitude of 200 Pa to allow the stress induced during sample loading to relax. The extent of the linear viscoelastic region (LVR) had been previously determined by performing stress sweep tests (20–2000 Pa) at 1 Hz. Additionally, frequency sweep tests were conducted by applying a constant sinusoidal stress of 200 Pa within the LVR over a frequency range between 0.1 and 10 Hz. The test temperature was consistently maintained at 20 °C. The rheological properties recorded for the study included the storage modulus (*G*′, kPa), loss modulus (*G*″, kPa), complex modulus (G*, kPa), and the strain and stress determining the LVR limit (*γ*_c_, %%; *σ*_c_, Pa). Furthermore, experimental data from frequency sweeps were fitted to a power law, known as the weak gel model [28] (Equation (1)):

G*(*f*) = [*G*′(*f*)^2^ + *G*″(*f*)^2^]^0.5^ = *Af*^1/z^ (1)

where G* is the complex modulus in kPa, *f* is the frequency in Hz, *A* (kPa s^1/z^) is the proportional coefficient or strength of the interactions (G* at 1 Hz), and z (dimensionless) is the coordination number or network extension. *α* = 1/z is also referred to as the order of relaxation function [29]. Measurements were conducted after 3, 18, 33, and 60 days of storage.

### 2.7. Laminating Fat (LF) Texture Measurement

The LF consistency over the chilling storage period was evaluated using a TA.HDPlus Texture Analyzer (Stable Micro Systems, Ltd., Godalming, Surrey, UK) equipped with a 5 kg load cell and provided with version 6.1.20.0 of Texture Exponent software. A penetration test was conducted using a 30° conical methacrylate probe, which penetrated the sample up to 10 mm at a deformation rate of 1 mm/s. The maximum force or firmness (N) and work or spreadability (mJ) at 10 mm were derived from the force–distance curves generated. Measurements were carried out at 20 °C after 3, 18, 33, and 60 days of storage.

### 2.8. Laminating Fat (LF) Oil-Binding Capacity and Colorimetric Analyses

The method of Da Pieve et al. [30] was adapted for calculating the oil-binding capacity (OBC) of all formulated LFs and the controls CB and CLF. For color evaluation, a Konica Minolta CR-400 ChromaMeter (Konica Minolta Sensing, Inc., Osaka, Japan) was used, and the color parameters brightness (L*), greenness (−a*), and yellowness (+b*) were determined by reflectance measured according to the Commission Internationale de l’Eclairage (CIE) L*a*b* uniform color space method. Measurements were performed for each sample after 3, 18, 33, and 60 days of storage.

### 2.9. Laminating Fat (LF) Lipid Oxidation

Secondary lipid oxidation was determined by thiobarbituric acid-reactive substances (TBARS) according to the method of Freire et al. [31], with some modifications, as described in Cofrades et al. [32]. The results are expressed as mg of malondialdehyde (MDA) per kilogram of sample (mg MDA/kg sample). Measurements were conducted for each LF after 3, 18, 33, and 60 days of storage.

### 2.10. Puff Pastry (PP) Baking Performance

For all four baked PPs made with the formulated LFs, the weight (W_p_) and thickness (T_p_) of each square paste or laminated dough piece with a fixed surface area (5 × 5 cm^2^) were recorded beforehand. To evaluate the quality of the PPs, after baking and cooling to room temperature, each baked PP piece counterpart was weighed (W_PP_), and its height (H_PP_) was measured. From these data, weight loss (%) and the lifting or puffing effect (%) were calculated as (W_p_ − W_PP_/W_p_) × 100 and (H_PP_ − T_p_/H_PP_) × 100, respectively, representing the relative PP weight loss and height increase during baking. In addition, the length (longer dimension) and width of the PPs were measured in order to estimate the two-dimensional size loss from the initial fixed surface area of the paste piece mentioned above. Dimensional measurements of all pastes and PP samples were obtained manually using an electronic caliper. The LF samples were used to bake the PPs after 3, 18, 33, and 60 days of storage.

### 2.11. Puff Pastry (PP) Texture Measurement

One hour after baking, the texture of the PPs at all four storage times was evaluated using the same TA.HDPlus Texture Analyzer mentioned above (Section 2.7.), equipped with the same 5 kg load cell. A texture profile analysis (TPA; double compression) test was also conducted on all the PP samples using a flat, circular aluminum compression plate (75 mm diameter, P/75; SMS Ltd., Surrey, UK). The contact force was 2 g, and the TPA test was performed at a compression rate of 1 mm/s up to a 30% strain. A 3 s waiting time was used between the first and second compressions. Hardness (N), cohesiveness (dimensionless), and chewiness (N) were obtained from the force–time profile [16]. Additionally, a cutting test was performed using a craft knife adapter (A/CKB; SMS Ltd., Godalming, Surrey, UK), which accommodated a standard 50 mm wide craft blade. Each PP piece was cut through its center up to a depth of 15 mm at a compression speed of 2 mm/s. The contact force was 5 g. The maximum force or peak firmness (N) was calculated as the highest peak of the force–distance curve.

### 2.12. Statistical Analysis

All LF samples and their respective PP counterparts were prepared in triplicate. Then, at least three measurements were taken from each replicate for the aforementioned determinations. All measurements obtained for each LF and its PP counterpart were evaluated periodically over a 2-month period (mostly after 3, 18, 33, and 60 days of chilling storage) and analyzed by one-way ANOVA. Significant differences between pairs of means were evaluated by the Tukey test with a 95% confidence interval (*p* < 0.05). The CB and CLF were not evaluated periodically over time because the thermal history and process conditions of these control samples are unknown to us. However, all determinations obtained for the equivalent first time point (3 days) in the controls CB and CLF, as well as in their PP counterparts, PPCB and PPCLF, are presented for comparison but were not included in the statistical analyses. Analyses were performed using the software IBM SPSS for Windows, Version 29.0 (IBM Corp., Armonk, NY, USA).

## 3. Results and Discussion

### 3.1. Proximate Compositions of LFs

Table 1 shows the proximate compositions determined after 3 days of storage for the four LF samples in comparison to the controls CB and CLF. The food components of the OPO-based LFs were consistent with their formulations and target compositions (Table S1). Differences (*p* < 0.05) in moisture and fat contents were found among samples LF1–LF4, with LF1 and LF2 having higher moisture and lower fat contents compared to LF3 and LF4. However, both the moisture and fat contents of all the LFs were within the range of the two controls, the CB and CLF. Protein was not detected in the control samples, whereas the low protein content observed in the LF samples could be due to the inclusion of Verolec non-GMO IP at 1.5% in their formulations (Table S1), which contain soybean lecithin.

Although the presence of milk fat and vegetable oils and fats in the CB and CLF increased the ash content, the variations found are of limited significance. Positively, the proximate compositions of these six LFs are quite similar to each other and therefore do not determine the PP performance during storage.

### 3.2. Thermal Behavior of LFs during Chilling Storage

**Figure 1:**
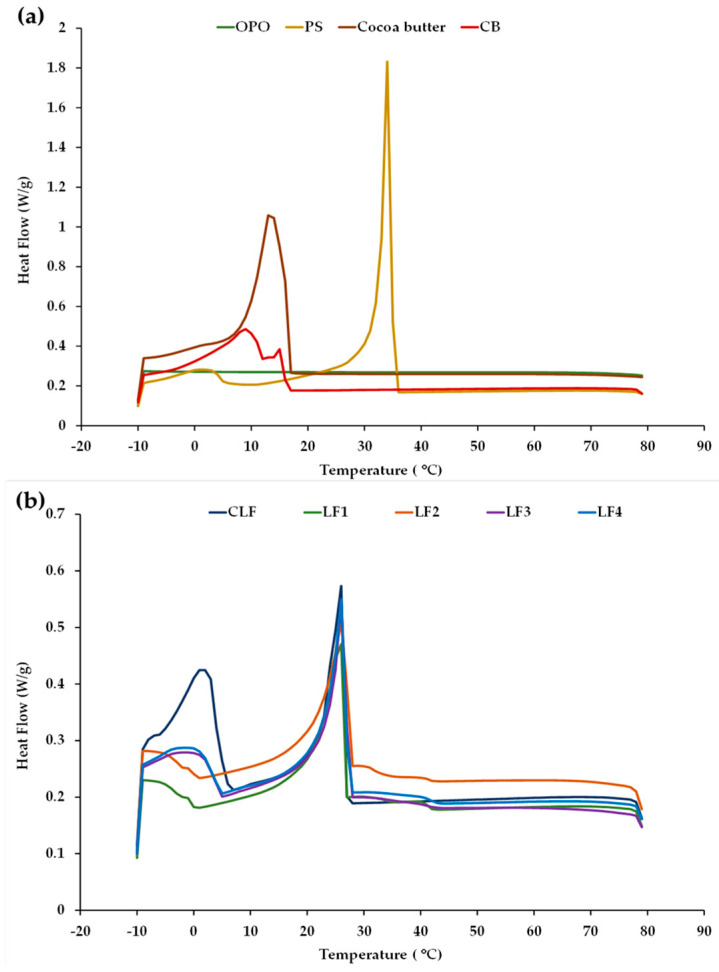
Cooling crystallization profiles obtained by DSC (**a**) for olive pomace oil (OPO), palm stearin (PS), cocoa butter, and a control commercial butter (CB) and (**b**) for a control commercial laminating fat (CLF) and laminating fats formulated with OPO (LF1–LF4) after 3 days of chilling storage.

Examples of the cooling thermograms obtained for LF1–LF4 after 3 days of storage, for the OPO, PS, and cocoa butter ingredients, and for the commercial controls CB and CLF are shown in Figure 1. For clarity, the profiles of OPO, PS, cocoa butter, and CB are grouped together (Figure 1a), while the profiles corresponding to the CLF are shown together with those obtained for the LF samples containing OPO (Figure 1b). No apparent differences in the cool flow patterns were detected at 60 days when compared to those observed after 3 days, suggesting sustained thermal stability for all the different LF samples during chilling storage under the given conditions.

No thermal events in the crystallization curve were observed in OPO (Figure 1a), reflecting the high thermal stability of this olive oil category between 80 and −10 °C. This stability is related to its high proportion of monounsaturated FAs (MUFAs) and low proportion of PUFAs [33]. In contrast, the palm oil present in the control CLF has a high SFA content and mainly consists of trisaturated PPP, disaturated POP, and monosaturated POO [34], which would justify the existence of the first principal exothermic peak in this oil at ~15–22 °C, associated with its high contents of saturated TAGs. For palm oil, POP is known to act as a crystal nucleator [9]. The crystallization curve of PS exhibited two characteristic peaks, although, in terms of sharpness and area, the first peak was much higher in magnitude (Figure 1a) and is indicative of major crystal formation due to the high content of palmitic FA and high-melting TAGs (POP and PPP) [35]. 

In turn, the cooling profile of cocoa butter exhibited a unique and distinct exothermic peak (Figure 1a), corroborating previous findings [36]. This peak is associated with its three main disaturated high-melting-point TAG species: POS (~40 mol%), 1,3-distearoyl-2-oleoyl-glycerol (SOS, ~28 mol%), and POP (~16 mol%) [37]. When compared to the other samples, in the control commercial butter (CB), homogeneous nucleation started at a lower temperature during cooling (Figure 1a), with a smaller first peak at around 15 °C and a second major peak at around 9 °C. The first peak is attributed to milk fat high-melting-point TAGs (crystallization-inducing agents), while the second peak is associated with medium- and low-melting-point TAGs [3,38].

Based on the similarity of the exothermic curves (Figure 1b), in the control CLF and formulated samples LF1–LF4, the first peak is evidently associated with their high PS content [9]. In addition, the cooling curves of the CLF, LF3, and LF4 (with both LFs having 31% OPO) exhibited a second crystallization peak at a low temperature, around 0–3 °C, whereas this second peak was not detected in samples LF1 and LF2, which did not contain cocoa butter.

On the other hand, Table 2 shows the crystallization and melting temperatures obtained for PS and cocoa butter, the controls CB and CLF, and the formulated samples LF1–LF4 after 3 and 60 days of chilling storage. PS exhibited two crystallization and three melting peaks. For PS, T_cp1_ (34.5 °C) aligns with the crystallization temperature range reported by other authors (24–40 °C) [8] and is associated with primary crystal formation, mainly by POP and PPP. It has been reported that PPP crystallizes rapidly in the β′ form at around 38 °C [39]. As expected, in the CLF and LF1–LF4, T_cp1_ shifted to a lower temperature when compared with neat PS (Table 2). There were no noticeable effects of the formulation or initial cooling rate on the T_cp1_ values of the four LF samples containing OPO, which ranged from 26.1 to 26.5 °C. The lower T_cp1_ value of the control CLF (24.9 °C) could be ascribed to a lower PS content when compared to the LF samples (PS at 23.5%; Table S1)—although this content is not indicated by the manufacturer on the CLF label—as well as to its high palm oil fraction content rich in POO.

With regard to the chilling storage effect studied, chilling time only had a significant effect (*p* < 0.05) on the T_cp1_ value of LF4, showing a decreased value after 60 days of storage compared to that observed after 3 days. In addition, after 3 days of storage, the initial cooling rate did not significantly affect the T_cp2_ values of LF3 and LF4, which were ~0.5 °C (Table 2), lower than the T_cp2_ values of both neat PS and the control CLF. Related to this fact, Salas Sotaminga et al. [40] indicated that the presence of a peak at around 5 °C could suggest the existence of metastable β′ polymorphs in these fat crystal networks, which are desirable for their small crystal size, which confers mouthfeel and spreadability [23,41]. In turn, chilling storage had a significant effect on the T_cp2_ values of LF3 and LF4, which were higher and around 1 °C after 60 days. This result indicates that, during 60-day storage at 4 °C, the solid fat content (SFC) of both LFs probably increased due to continued TAG crystallization and/or the ripening or aggregation of existing crystals at low temperatures [41].

Regarding the melting points (Table 2), PS, CB, and the formulated samples LF1–LF4 exhibited three melting peak temperature (T_mp_) values.

The higher T_mp3_ for hard PS (54.6 °C) is due to the melting of the PPP crystals mentioned above. In the control CB (Table 2), milk fat exhibited three melting peaks, with the maximum peak temperature (T_mp3_) at 33.7 °C and the second peak (T_mp2_) at approximately 14 °C, corresponding to the middle-melting TAG fraction [42]. Compared to the CB, in the control CLF and in the LF samples formulated with OPO, the blend of milk fat with PS resulted in a significant increase in the T_mp3_ value due to the very high-melting characteristics of PS. In addition, the CLF exhibited a fourth event at a high melting temperature (T_mp4_ at 47.3 °C).

On the other hand, chilling storage had a significant effect (*p* < 0.05) on the T_mp1_ values of LF1, LF3, and LF4, as well as on the T_mp3_ values of LF2, LF3, and LF4 (Table 2), which increased and decreased, respectively, during the storage period. These modifications likely reflect undesired post-crystallization processes occurring during low-temperature storage, with more significant effects being observed in LF3 and LF4 (31% OPO and cocoa butter) as compared to LF1 and LF2 (41% OPO). However, the two different initial crystallization conditions used did not affect the crystallization and melting peak temperatures of either LF3 and LF4 or LF1 and LF2. The enthalpy requirements associated with each peak during the cooling and melting stages, i.e., the amount of energy lost and gained during the crystallization and melting processes, respectively [9], can be consulted in Table S2.

### 3.3. Rheological and Textural Behaviors of LFs during Chilling Storage

#### 3.3.1. Dynamic Shear Properties and Weak Model Parameters

**Figure 2:**
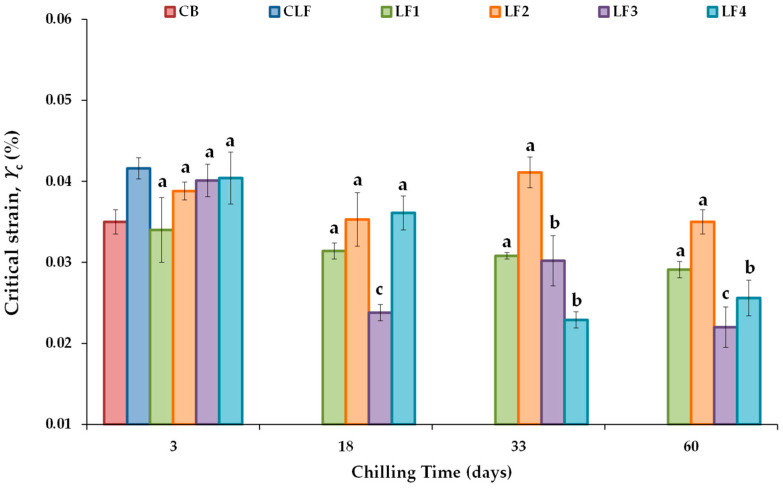
Critical strain (γ_c_) values from stress sweep tests for the different laminating fats tested during chilling storage; shear stress ranged between 20 and 2000 Pa at 1 Hz and 20 °C. CB, control commercial butter; CLF, control commercial laminating fat; LF1–LF4, laminating fats formulated with olive pomace oil (OPO). Different letters for the same LF during chilling storage indicate significant differences (*p* < 0.05).

Figure 2 shows the critical strain (*γ*_c_) value, defined as the strain above which the complex modulus (G*) decreases by more than 10% of its initial constant value [43], ensuring the linear viscoelasticity of all the LF samples tested during chilling storage. The controls CB and CLF exhibited *γ*_c_ values (%) of 0.0350 ± 0.0015 and 0.0416 ± 0.0013, respectively. No significant differences in *γ*_c_ values were found between the samples containing 41% OPO (LF1 and LF2) and 31% OPO with 10% cocoa butter (LF3 and LF4) due to the initial cooling rate effect. For the formulated LFs, the *γ*_c_ values after 3 days of storage ranged between 0.0340 ± 0.0040 and 0.0404 ± 0.0032, with the lowest and highest values corresponding to LF1 and LF4, respectively. Furthermore, after 3 days of storage, the associated critical stress (*σ*_c_) values were 356 ± 18 Pa in LF1 and LF2 and 399 ± 7.5 Pa in LF3 and LF4. These values conform to the requirement that an ideal fat mimetic should have a yield stress between 200 and 1000 Pa [44].

Regarding the effect of chilling storage, significant changes in *γ*_c_ did not occur over the storage period studied in LF1 and LF2 (Figure 2). However, in both LF3 and LF4, *γ*_c_ values were significantly lower after 33 and 60 days of storage than after 3 days. *γ*_c_ is considered a measure of the network conformational flexibility [43], as well as an index of emulsion extensibility [45]. In LF3 and LF4, conformational flexibility decreased with increasing storage time, indicating that in the presence of cocoa butter, there would be a potential limit of storage days at 4 °C (18 and 33 days for LF3 and LF4, respectively; Figure 2). This limitation is likely due to the increased density of the entanglement network, which results in an increase in the brittleness consistency as well. Although initially (after 3 days), all LF samples with OPO had a similar network, LF3 and LF4 contain cocoa butter, which is known to agglomerate and form large crystal structures in palm oil blends [7], thereby increasing the gel strengths of these margarines. This decrease in extensibility adversely affected the ability of LF3 and LF4 to spread without breaking during the paste-making procedure and, therefore, their baking performance, as described below.

According to the weak gel model proposed by Gabriele et al. [28], the rheological structure of LF samples can be considered a three-dimensional network in which droplet particles and similar units are linked by weak and strong interactions. As an example, Figure S1 shows the frequency sweep results for G* of the LF samples after 60 days of storage compared to the controls CB and CLF, along with the fits to the weak gel model proposed by Gabriele et al. [28]. Similar G* vs. *f* plots were obtained for earlier storage times. From the logarithmic-scale graph, potential regression fits allow the direct determination of the proportional coefficient (*A*) value, whereas the coordination coefficient (*z*) is derived as the reciprocal of the frequency exponent.

Over the storage period studied, the magnitudes of the *R*^2^ fits were very close to 1 (>0.990), demonstrating the adequacy of the weak gel model to describe the rheological structure of the LF systems studied. A rheological characterization based on the parameters *z* and *A* was also useful in distinguishing mayonnaises made with different alginate concentrations [45] and water-in-oil structured emulsions and margarines with varying olive oil/cocoa butter proportions [46]. Note that the frequency dependence of G* for LF1 and LF2 was lower than for LF3, LF4, and both controls CB and CLF, as indicated by their lower 1/z exponent values (Figure S1).

**Figure 3:**
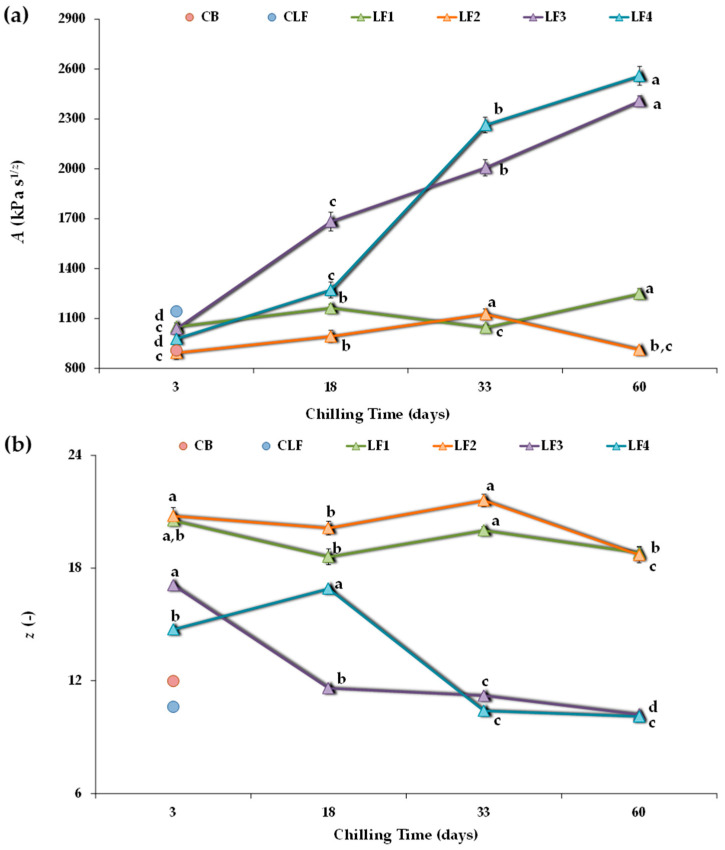
Power-law parameters describing frequency sweeps for the different laminating fats tested during chilling storage. (**a**) *A*, proportionality coefficient (G* at 1 Hz); (**b**) *z*, coordination number. CB, control commercial butter; CLF, control commercial laminating fat; LF1–LF4, laminating fats formulated with olive pomace oil (OPO). Different letters for the same LF during chilling storage indicate significant differences (*p* < 0.05).

Figure 3 shows the *A* and *z* values obtained for all four experimental design samples. After 3 days of storage, LF2 had a lower *A* value than LF1 and LF3, although the differences between samples were not relevant. However, both LF1 and LF2, containing 41% OPO, presented notably higher *z* values (20.5 ± 0.27 and 20.8 ± 0.45, respectively) than LF3 and LF4 (17.1 ± 0.14 and 14.7 ± 0.094, respectively), suggesting the presence of a denser network in LF1 and LF2. In addition, LF4, which initially cooled faster than LF3, showed a significantly lower *z* value. Furthermore, after 3 days of storage, all formulated LFs exhibited comparable network strength (*A* value) to the controls CB and CLF (Figure 3a), although with much higher *z* values (Figure 3b). It is interesting to note that the coordination number (*z*) was 12.0 ± 0.24 in the CB and that the CLF had the lowest number of interacting rheological units within a more extended three-dimensional network (10.6 ± 0.12). A distinct elastic character was associated with an order of relaxation function α (=1/*z*) around 0.1, as observed in the CLF (Figure S1).

Regarding the studied effect of chilling storage time on each formulated LF, it is possible to observe that with increasing storage time, the model parameters changed more for LF3 and LF4 than for LF1 and LF2 (Figure 3). In both LF3 and LF4, a pronounced strengthening of the network occurred, and the *A* value significantly (*p* < 0.05) increased over time (Figure 3a) because interaction forces within the network increased [45]. In fact, for these LFs containing cocoa butter and lower OPO (31%) content, the *A* values after 60 days of storage were more than 2-fold higher than those after 3 days. However, although storage time also significantly affected the strength of the interactions in LF1 and LF2, there were no significant differences between the *A* values of LF1 after 3 and 33 days of storage, nor between those of LF2 after 3 and 60 days. Thus, the structural changes occurring during storage were mitigated by using OPO at a 41% concentration and without cocoa butter in the LF formulation.

In turn, in LF3 and LF4, increasing the storage time weakened the network connectivity (decreasing the *z* value) (Figure 3b). This effect was less pronounced in LF1 and LF2, where the number of interacting units cooperating in the network tended to remain more constant over time, although higher than in the controls CB and CLF, both initially and throughout the storage period. On the contrary, for LF3 and LF4, after 33 and 60 days of storage, the *z* coordination number decreased, reaching values comparable to those of the controls CB and CLF (Figure 3b). However, this reduction in *z* always correlated with much higher *A* values (Figure 3a). Indeed, the decrease in *z* could be associated with aggregation phenomena, where larger particles form a less interconnected network [7].

Therefore, post-hardening during storage occurred for all the LFs during storage at 4 °C. This was accompanied by an increase in the strength of the interactions between the rheological units. However, post-hardening was much more significant in samples LF3 and LF4, which included cocoa butter and a lower OPO content.

Therefore, the dynamic rheological measurements indicate that while the appearance of aggregates during storage was evident for all formulated LF samples, it seemed to occur much more slowly in samples LF1 and LF2. Indeed, although the data are not included, substantial hardening was also detected for the CLF, reaching *A* and *z* values of 4372 kPa and 13.2, respectively, after two months at 4 °C, thus corroborating previous findings [7,8,10,11]. In contrast, this phenomenon was not detected in the control commercial butter (CB).

#### 3.3.2. Textural Parameters

**Figure 4:**
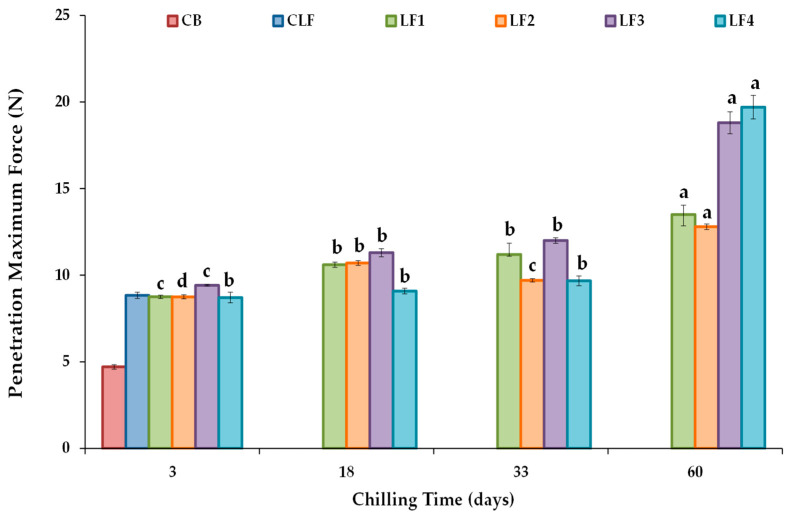
Conical penetration maximum force or firmness values at 20 °C for the different laminating fats tested during chilling storage. CB, control commercial butter; CLF, control commercial laminating fat; LF1–LF4, laminating fats formulated with olive pomace oil (OPO). Different letters for the same LF during chilling storage indicate significant differences (*p* < 0.05).

The maximum force or firmness values derived from a conical penetration test are shown in Figure 4. After 3 days of storage, LF3, which initially cooled at a slower rate than LF4, exhibited higher firmness (9.41 ± 0.045 N) than LF1 (8.75 ± 0.098 N), LF2 (8.75 ± 0.12 N), and LF4 (8.71 ± 0.31 N). However, differences between samples due to composition were not considered relevant, and the initial cooling rate did not modify the firmness of samples with higher OPO content. In turn, the firmness value of the control CLF was 8.83 ± 0.18 N, very similar to those of LF1, LF2, and LF4 after 3 days of storage. Nonetheless, the CB required much less force for deformation (4.70 ± 0.14 N), as milk fat contains a significantly lower SFC (6–20%) at 20 °C than the vegetable LF samples [42].

The change in firmness values during storage for each formulated LF is shown (Figure 4). Significant increases (*p* < 0.05) were observed for all the LFs, particularly after 60 days of storage compared to the values measured after 3 days, although formulations LF3 and LF4 showed much higher firmness than LF1 and LF2. Between days 3 and 60 of storage, firmness increased by approximately 50% for LF1 and LF2 and by about 100% and 125% for LF3 and LF4. Various solutions have been proposed to address post-crystallization issues during margarine storage, such as controlling the diacylglycerol (DAG) content to increase crystallization rates, as well as employing specific processing methods [47]. In this study, the content of emulsifiers was fixed and constant in all the formulated LF samples (Table S1), and the heightened hardening observed in LF3 and LF4 can be attributed to the presence of cocoa butter and the lower OPO content in these formulations. Interestingly, although LF4 showed the highest increase in firmness after 60 days (19.7 ± 0.68 N), only in this LF did the force required for deformation remain constant throughout the first 33 days of storage (Figure 4).

The increase in firmness was also associated with a rise in the work value or spreadability during storage (Figure S2), with percentage increases of ~47, 41, 100, and 119% for LF1, LF2, LF3, and LF4 when comparing days 3 and 60 of storage. A higher work value indicates a higher difficulty degree to spread the margarine as a thin and uniform layer [48]. Therefore, especially after 60 days of storage, the formulated samples LF1 and LF2 were more spreadable than LF3 and LF4. The importance of the polymorphic nature of the fat crystals in perceived spreadability has also been indicated [49].

### 3.4. Aggregate Formation in LFs during Chilling Storage

**Figure 5:**
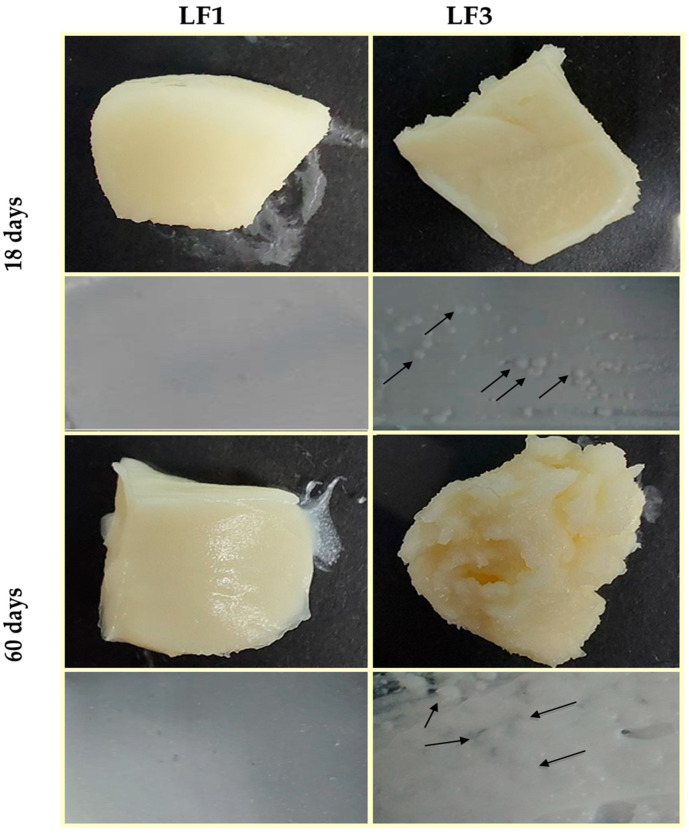
Images of laminating fats (LF1 and LF3) after 18 and 60 days of chilling storage at 4 °C. Granular crystals formed in LF3 are indicated with arrows.

Figure 5 shows images of the LF pieces and LF spreads prepared with 41% OPO (LF1) and with 31% OPO and 10% cocoa butter (LF3) after 18 and 60 days of chilling storage. Similar images were obtained for samples LF2 and LF4.

It can be observed that LF1 maintained a homogeneous and smooth appearance throughout the storage period studied, while in LF3, small aggregates were clearly noticeable after just 18 days of storage at 4 °C. In addition, in LF3, the formation and size of fat aggregate clusters increased with storage time (Figure 5), indicating quality deterioration during the storage of samples LF3 and LF4, which contained cocoa butter. Therefore, once again, it is reasonable to consider that the formation of these aggregates in LF3/LF4, responsible for the differences observed in their physicochemical properties, can be solely attributable to the presence of cocoa butter in these LF formulations.

Indeed, it is thought to be possible that OPO and cocoa butter are immiscible or, alternatively, that cocoa butter and PS are immiscible solid components. POO is one of the major TAGs found in OPO [19], while cocoa butter and PS are rich in symmetric POP [37,50] and symmetric PPP and POP TAGs [9], respectively. It has been reported that POP and PPP TAGs have a β tendency, while asymmetric POO TAGs remain stable in their β′ form [8]. In addition, Detry et al. [7] also reported that a high POP content implies slow crystallization, allowing a significant amount of fat to continue crystallizing during the storage of margarine, leading to the formation of the observed aggregates in LF3 and LF4. Interestingly, despite the apparent incompatibility between OPO and cocoa butter detected in this study, OPO has been successfully employed for cocoa butter-like fat production using *sn*-1,3-specific lipase to reduce the costs of confectionery products containing cocoa butter [50].

### 3.5. Oil-Binding Capacity, Color, and Lipid Oxidation of LFs during Chilling Storage

Over time, the oil-binding capacity (OBC) of all four formulated LFs was 100%, proving that the structuring agents (type and concentration) used in LF preparation (Table S1) led to the formation of an effective network, similar to that of both controls CB and CLF. Figure S3 illustrates the color of the LF samples during chilling storage. In comparison to the CB and CLF, the luminosity values (L*) of the LF samples were lower, the a* values were not significantly different, especially when compared with the control CB, and the b* values were also lower. After 3 days of storage, LF2 had lower L* and b* values and a more negative a* value than the rest of formulated LF samples; however, these variations are of very limited importance. All LF samples in this study exhibited a greenish-yellow color, and some were more opaque than the controls. The color of LFs and margarines is usually dependent on the composition and the liquid oil used, and LF1 and LF2 contained more OPO than LF3 and LF4. In this study, the natural color was measured; however, it is worth noting that the color of the shortenings can be easily modified by adding oil-soluble colorants to OPO [51].

On the other hand, the color of the LF samples remained relatively stable during the chilling storage period (Figure S3). For instance, the values of the color parameters in sample LF1 showed ranges of variation from 80.8–83.6 for L*, −2.76 to −3.59 for a*, and 17.0–22.1 for b*, while those of sample LF3 were 81.7–85.2 for L*, −2.28 to −3.27 for a*, and 15.7–22.7 for b*. However, in most cases, the yellowness of the formulated LF samples tended to decrease at the end of the storage period. One of the characteristics of degraded margarine is its discoloration induced by oxidation [52]. Nonetheless, the authors just cited demonstrated that the use of a biodegradable/active smart nano-composite film containing polylactic acid, titanium dioxide, and lycopene nanoparticles increased the color and oxidative stability of margarine during storage. Figure S4 shows the lipid oxidation experienced by the four LF samples during the 2-month chilling storage period in comparison to the controls CB and CLF. The TBARS value is commonly used to indicate the occurrence of the second stage of lipid auto-oxidation [53]. In the formulated LF samples, the TBARS value was scarcely influenced by the formulation and initial cooling rate. After 3 days of storage, all LF samples presented relatively low TBARS values (ranging between 1.07 and 1.14 mg MDA/kg sample), and the product with the highest oxidation value from the beginning of the study was the control CLF (3.23 mg MDA/kg sample). This result indicates lipid oxidation in the CLF, possibly associated with its unknown previous thermal history and with the storage conditions of the study (4 °C), as the manufacturer recommends higher storage temperatures (between 12 and 18 °C). In addition, the CLF is rich in UFAs [16], which makes it susceptible to oxidation, leading to rancidity. In contrast, the control CB had the lowest TBARS value (0.215 mg MDA/kg sample), as expected, given its high SFA and low UFA contents.

On the other hand, despite the high UFA content of LF1–LF4 [16] and the fact that no special antioxidant packaging material was used in this study, chilling storage had no significant effect (*p* > 0.05) on all four formulated LF samples, with TBARS values below 1.5 mg MDA/kg sample. The maximum acceptable TBARS value in oils may vary depending on the food product, but it is generally between 1.5 and 2.0 mg MDA/kg sample [53].

### 3.6. Performance of Puff Pastry during Chilling Storage

**Figure 6:**
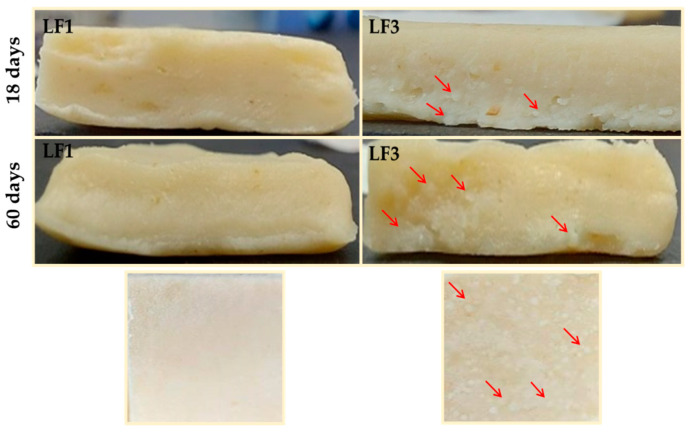
Images of pastes or laminated doughs prepared with laminating fats (LF1 and LF3) after 18 and 60 days of chilling storage at 4 °C. Granular crystals formed in the paste with LF3 are indicated with arrows.

Figure 6 shows images of the laminated dough or paste counterparts prepared from samples LF1 and LF3 shown in Figure 5. As expected, the granular crystals observed in LF3 after 18 and 60 days of chilling storage were also noticeable in the pastes laminated with it.

Starting from day 18 of storage, and because of its hard and brittle character (Figure 4), both LF3 and LF4 were very difficult to handle during the sheeting and folding steps of the lamination process. Inevitably, the harder LF samples containing fat aggregates (Figure 5) caused damage to and the breakage of the dough layers, resulting in a non-homogeneous layering, with large fat crystals also being present in the pastes (Figure 6). On the contrary, both LF1 and LF2 formed very smooth and homogeneous laminated doughs throughout the entire storage period studied.

The effect of chilling storage on the performance of the baked PPs prepared with the controls CB and CLF and samples LF1–LF4 is presented in Table 3. In terms of weight loss, although significant differences were observed, they were relatively minor, with variations in this parameter ranging from 21.5 to 27.0%. Samples PP1 and PP2 exhibited higher weight losses after 33 and 60 days of storage compared to those registered after 3 and 18 days. In contrast, this parameter remained almost constant in samples PP3 and PP4, despite already being high at the beginning of chilling storage. As for the height of the PP samples, the laminated doughs made with LF1 and LF2 consistently presented greater height. Furthermore, at the end of the storage period, the height remained constant in PP1 and was even higher in PP2 when compared to that observed after 3 days of storage (Table 3). On the contrary, in the PPs prepared with the LF samples with lower OPO content and cocoa butter (samples PP3 and PP4), a significant decrease in height was already noticed after 18 days of storage. This reduced height in the PP is associated with the breakage and presence of clusters of dough layers in the pastes (Figure 6), which favors the escape of steam and prevents the pastry from expanding. The lifting or puffing effect was consistently more pronounced in PP1 and PP2 than in PP3 and PP4, even increasing after 60 days of storage in the former and decreasing in the latter, compared to measurements obtained after 3 days.

Despite the fact that the PP length and width remained almost constant throughout chilling storage in the PPs made with all four LF samples (Table 3), it is evident that the storage time had a more negative effect on the baking performance of products made with the LF samples that contained cocoa butter and less OPO.

### 3.7. Texture of Puff Pastries during Chilling Storage

Table 4 illustrates the properties and textural parameters derived from TPA and cutting tests conducted on PP1–PP4 prepared with LF1–LF4 during chilling storage in comparison with the PPs made with the commercial controls CB and CLF (PPCB and PPCLF). Note that after 3 days of storage, the four PPs formulated with the LFs containing OPO already exhibited higher hardness and chewiness than PPCB and PPCLF. Notably, PP1 and PP2 maintained their hardness and chewiness almost consistently until the end of the storage period (60 days). On the one hand, this result indicates the stability of the crystal network structure of the LF samples containing 41% OPO (LF1 and LF2), regardless of the initial crystallization temperature used. On the other hand, this suggests that there is no loss of functionality during baking over the entire 60-day storage period, as previously described.

In contrast, samples PP3 and PP4 showed a very significant increase in hardness and chewiness after 18 days of storage, with a hardness value almost 4 times higher than that recorded after 3 days (Table 4). In addition, after 60 days, samples PP3 and PP4 were considerably harder and much more difficult to chew and, therefore, had a more difficult workability and worse texture than samples PP1 and PP2, formulated without cocoa butter.

Likewise, the values of the maximum force required to cut the four PP samples were quite similar to those at the beginning of storage and remained practically constant until the end of the storage period in the case of PP1 and PP2. In contrast, firmness increased significantly in samples PP3 and PP4. Again, these textural modifications observed are connected to post-crystallization processes [7], which were markedly pronounced during the storage of the LF samples containing cocoa butter in addition to OPO (Figure 5 and Figure 6).

## 4. Conclusions

Four LF or margarine samples were formulated, two containing 41% OPO (LF1 and LF2, with slower and faster initial cooling rates, respectively) and two containing 31% OPO and 10% cocoa butter (LF3 and LF4, with similar static crystallization conditions to LF1 and LF2, respectively), to study their stability during chilling storage conditions. Changes in the crystallization and melting temperatures of samples LF3 and LF4 indicate undesired post-crystallization processes and rearrangements in the crystal structures during the chilling storage. The rheological measurements in LF3 and LF4 also showed a decrease in conformational flexibility with increasing storage time, which was accompanied by a pronounced strengthening of the network due to increased interaction forces within the network. Post-hardening during storage at 4 °C was much more significant for samples LF3 and LF4, which had lower OPO content and cocoa butter, than for samples LF1 and LF2. During LF3 and LF4 chilling storage, some fat aggregates were formed. Furthermore, due to their hard and brittle character, both LF3 and LF4 were very difficult to handle during the sheeting and folding steps of the lamination process starting from day 18 of storage. In contrast, both LF1 and LF2 formed very smooth and homogeneous laminated doughs throughout the entire storage period studied. As a consequence, the baking performance remained constant in samples LF1 and LF2 during chilling storage, whereas a significant height loss was already observed after 18 days of storage in the PP3 and PP4 made with LF3 and LF4. Therefore, the mixing of OPO with cocoa butter should be avoided to maintain the quality and stability of OPO-based margarines for multilayered products. In addition, regarding processing conditions, it would be appropriate to test LF1 and LF2 sample production at both the pilot and industrial scales to optimize crystallization.

## Figures and Tables

**Table 1:** Proximate compositions of a commercial butter (CB), a commercial laminating fat (CLF), and four formulated laminating fats (LF1–LF4) containing olive pomace oil (OPO).

Sample	Moisture (g/100 g)	Protein (g/100 g)	Ash (g/100 g)	Fat (g/100 g)
CB	14.5 ± 0.15	nd	0.131 ± 0.011	85.4 ± 0.11
CLF	18.8 ± 0.15	nd	0.805 ± 0.015	80.4 ± 0.17
LF1	16.7 ± 0.12 ^a^	0.486 ± 0.076 ^a^	1.24 ± 0.0093 ^a^	81.6 ± 0.15 ^c^
LF2	17.0 ± 0.19 ^a^	0.486 ± 0.076 ^a^	1.24 ± 0.0093 ^a^	81.3 ± 0.14 ^c^
LF3	14.8 ± 0.20 ^c^	0.454 ± 0.0034 ^a^	1.28 ± 0.041 ^a^	83.5 ± 0.15 ^a^
LF4	15.6 ± 0.0038 ^b^	0.454 ± 0.0034 ^a^	1.28 ± 0.041 ^a^	82.7 ± 0.12 ^b^

Mean values (*n* = 3) ± standard deviation; nd, not detected. ^a–c^ Effect of formulation and initial cooling rate. Different letters in the same column for the same determination indicate significant differences (*p* < 0.05).

**Table 2:** Crystallization and melting peak temperatures measured by DSC for the different laminating fats (LFs) containing olive pomace oil (OPO) tested at the beginning and end of the chilling storage period in comparison with the ingredients PS and cocoa butter and the controls CB and CLF.

Sample	Chilling Time (Days)	T_cp1_ (°C)	T_cp2_ (°C)	T_mp1_ (°C)	T_mp2_ (°C)	T_mp3_ (°C)	T_mp4_ (°C)
OPO	-	-	-	-	-	-	-
PS	-	34.5 ± 0.068	2.18 ± 0.29	7.54 ± 0.036	43.0 ± 0.13	54.6 ± 0.10	-
Cocoa Butter	-	13.5 ± 0.14	-	20.5 ± 0.030	-	-	-
CB	-	15.3 ± 0.056	8.64 ± 0.46	8.40 ± 0.098	15.2 ± 0.023	33.3 ± 0.24	-
CLF	-	24.9 ± 1.1	2.47 ± 0.20	2.56 ± 0.050	8.33 ± 0.030	41.8 ± 0.12	47.3 ± 0.070
LF1	3	26.3 ± 0.41 ^a^	-	−3.20 ± 0.11 ^b^	3.44 ± 0.30 ^a^	46.3 ± 0.87 ^a^	-
LF2	3	26.2 ± 0.13 ^a^	-	−2.75 ± 0.12 ^a^	3.54 ± 0.15 ^a^	46.6 ± 0.37 ^a^	-
LF3	3	26.1 ± 0.20 ^a^	0.470 ± 0.092 ^b^	0.150 ± 0.00 ^b^	7.31 ± 0.076 ^a^	45.8 ± 0.16 ^a^	-
LF4	3	26.5 ± 0.16 ^a^	0.493 ± 0.025 ^b^	0.290 ± 0.026 ^b^	7.25 ± 0.20 ^a^	45.9 ± 0.24 ^a^	-
LF1	60	26.2 ± 0.35 ^a^	-	−2.88 ± 0.14 ^a^	3.53 ± 0.055 ^a^	45.9 ± 0.025 ^a^	-
LF2	60	26.0 ± 0.050 ^a^	-	−2.73 ± 0.19 ^a^	3.39 ± 0.13 ^a^	45.3 ± 0.43 ^b^	-
LF3	60	26.3 ± 0.19 ^a^	1.04 ± 0.12 ^a^	0.790 ± 0.044 ^a^	7.32 ± 0.20 ^a^	44.7 ± 0.16 ^b^	-
LF4	60	25.9 ± 0.13 ^b^	0.763 ± 0.045 ^a^	0.733 ± 0.015 ^a^	6.98 ± 0.12 ^a^	44.6 ± 0.061 ^b^	-

Mean values (*n* = 3) ± standard deviation. DSC, differential scanning calorimetry; OPO, olive pomace oil; PS, palm stearin; CB, control commercial butter; CLF, control commercial laminating fat; LF1–LF4, laminating fats formulated with OPO; T_cp1_ and T_cp2_, peak temperatures measured from crystallization thermograms; T_mp1_, T_mp2_, T_mp3_, and T_mp4_, peak temperatures measured from melting thermograms. (-), chilling time not available or no peak temperature detected; ^a,b^ Different letters in the same column and for the same LF indicate significant differences (*p* < 0.05).

**Table 3:** The effect of chilling storage on the performance of puff pastries (PPs) produced with laminating fats (LFs) containing OPO in comparison with PPs made with the CB and CLF.

Puff Pastry (PP)	Chilling Time (Days)	Weight Loss (%)	PP Height (mm)	Lifting (%)	PP Length (mm)	PP Width (mm)
PPCB	-	20.5 ± 1.3	44.1 ± 3.6	661 ± 61	46.2 ± 2.6	42.8 ± 2.1
PPCLF	-	22.7 ± 3.2	40.1 ± 4.4	592 ± 75	45.1 ± 3.6	41.1 ± 3.3
PP1	3	23.2 ± 1.2 ^b^	30.1 ± 3.8 ^a,b^	484 ± 75 ^b^	48.6 ± 5.8 ^a^	41.2 ± 5.6 ^a^
	18	22.4 ± 1.0 ^b^	27.3 ± 2.9 ^b,c^	371 ± 50 ^c^	46.6 ± 2.6 ^a,b^	40.5 ± 3.7 ^a^
	33	25.4 ± 1.6 ^a^	22.0 ± 2.0 ^c^	286 ± 36 ^d^	44.4 ± 2.5 ^b^	39.9 ± 2.2 ^a^
	60	25.8 ± 1.4 ^a^	31.3 ± 2.7 ^a^	543 ± 55 ^a^	48.9 ± 1.3 ^a^	40.6 ± 1.9 ^a^
PP2	3	23.1 ± 1.2 ^b^	23.6 ± 3.9 ^c^	345 ± 73 ^c^	44.1 ± 5.6 ^b^	40.2 ± 5.8 ^a^
	18	22.7 ± 2.5 ^b^	37.3 ± 2.9 ^a^	559 ± 52 ^a^	48.6 ± 2.4 ^a^	40.7 ± 3.2 ^a^
	33	27.0 ± 1.4 ^a^	27.5 ± 2.7 ^b^	438 ± 53 ^b^	45.7 ± 2.4 ^a,b^	38.2 ± 2.6 ^a^
	60	27.4 ± 1.2 ^a^	30.6 ± 3.4 ^b^	524 ± 68 ^a^	47.6 ± 1.9 ^a,b^	37.3 ± 1.2 ^a^
PP3	3	25.7 ± 0.76 ^a^	27.4 ± 2.8 ^a^	394 ± 50 ^a^	47.6 ± 4.1 ^a,b^	41.2 ± 3.5 ^a^
	18	21.5 ± 5.6 ^b^	18.6 ± 2.0 ^b,c^	220 ± 34 ^c^	47.8 ± 2.0 ^a^	42.5 ± 2.2 ^a^
	33	25.5 ± 4.8 ^a^	19.9 ± 1.3 ^b^	296 ± 26 ^b^	45.2 ± 1.9 ^b^	41.2 ± 1.4 ^a^
	60	24.7 ± 1.4 ^a,b^	17.3 ± 1.3 ^c^	235 ± 26 ^c^	47.8 ± 1.8 ^a^	43.0 ± 2.7 ^a^
PP4	3	25.6 ± 0.83 ^a,b^	24.8 ± 3.0 ^a^	340 ± 53 ^a^	48.2 ± 3.2 ^a^	42.5 ± 4.2 ^a^
	18	25.0 ± 1.6 ^a,b^	17.1 ± 1.3 ^c^	223 ± 25 ^c^	44.6 ± 2.1 ^b^	41.9 ± 1.6 ^a,b^
	33	24.0 ± 4.0 ^b^	21.7 ± 1.6 ^b^	276 ± 28 ^b^	46.2 ± 1.3 ^a,b^	42.3 ± 2.4 ^a,b^
	60	26.3 ± 1.5 ^a^	17.8 ± 1.4 ^c^	232 ± 26 ^c^	47.5 ± 2.2 ^a^	40.1 ± 1.6 ^b^

Mean values (*n* = 13–18) ± standard deviation. PP-CB, PP produced with a commercial butter; PP-CLF, PP produced with a commercial laminating fat; PP-LF1, PP-LF2, PP-LF3, and PP-LF4, PPs produced with LFs containing olive pomace oil (OPO). ^a–c^ Different letters in the same column and for the same PP indicate significant differences (*p* < 0.05).

**Table 4:** Effect of chilling storage on the textural properties from texture profile analysis (TPA) tests and maximum force from cutting tests for puff pastries (PPs) produced with laminating fats (LFs) containing OPO in comparison with PPs produced with the CB and CLF.

Puff Pastry (PP)	Chilling Time (Days)	Hardness (N)	Cohesiveness (-)	Chewiness (N)	Maximum Force (N)
PPCB	-	3.62 ± 0.28	0.248 ± 0.023	0.525 ± 0.086	2.80 ± 0.31
PPCLF	-	3.79 ± 0.35	0.228 ± 0.018	0.387 ± 0.051	4.32 ± 0.55
PP1	3	6.58 ± 1.2 ^a^	0.220 ± 0.035 ^b^	0.625 ± 0.18 ^b^	5.15 ± 0.075 ^b^
	18	5.75 ± 0.66 ^a^	0.206 ± 0.0058 ^b^	0.521 ± 0.083 ^b^	4.27 ± 0.52 ^b,c^
	33	6.56 ± 0.67 ^a^	0.284 ± 0.013 ^a^	0.971 ± 0.089 ^a^	6.58 ± 0.56 ^a^
	60	6.17 ± 0.18 ^a^	0.177 ± 0.095 ^b^	0.553 ± 0.037 ^b^	3.66 ± 0.10 ^c^
PP2	3	8.61 ± 0.91 ^a^	0.243 ± 0.016 ^a^	0.856 ± 0.24 ^a^	8.18 ± 1.2 ^a^
	18	6.28 ± 0.37 ^b^	0.219 ± 0.019 ^a^	0.728 ± 0.033 ^b^	3.85 ± 0.20 ^b^
	33	7.32 ± 0.72 ^b^	0.167 ± 0.0024 ^b^	0.528 ± 0.023 ^b^	4.68 ± 0.54 ^b^
	60	7.37 ± 0.35 ^b^	0.158 ± 0.020 ^b^	0.466 ± 0.079 ^b^	5.58 ± 0.70 ^b^
PP3	3	8.72 ± 0.77 ^d^	0.235 ± 0.018 ^b,c^	1.16 ± 0.11 ^d^	6.86 ± 1.2 ^c^
	18	33.6 ± 0.74 ^b^	0.294 ± 0.041 ^a,b^	4.60 ± 0.82 ^b^	18.4 ± 0.94 ^b^
	33	23.8 ± 0.66 ^c^	0.222 ± 0.017 ^c^	2.96 ± 0.086 ^c^	17.1 ± 0.46 ^b^
	60	52.8 ± 1.3 ^a^	0.323 ± 0.011 ^a^	10.7 ± 0.43 ^a^	27.3 ± 1.3 ^a^
PP4	3	10.6 ± 0.89 ^c^	0.223 ± 0.0035 ^b^	1.18 ± 0.13 ^c^	7.21 ± 1.1 ^c^
	18	40.2 ± 0.063 ^a^	0.352 ± 0.0053 ^a^	6.03 ± 0.41 ^a^	19.1 ± 1.5 ^a^
	33	21.8 ± 1.1 ^b^	0.218 ± 0.012 ^b^	2.19 ± 0.24 ^c^	14.8 ± 0.52 ^b^
	60	37.8 ± 0.90 ^a^	0.249 ± 0.031 ^b^	4.74 ± 0.63 ^b^	21.7 ± 1.5 ^a^

Mean values (*n* = 3) ± standard deviation. PPCB, PP produced with a commercial butter; PPCLF, PP produced with a commercial laminating fat; PP1, PP2, PP3, and PP4, PPs produced with LFs containing olive pomace oil (OPO). ^a–d^ Different letters in the same column and for the same PP indicate significant differences (*p* < 0.05).

## Data Availability

Data are contained within the article or Supplementary Materials.

## Supplementary Materials

The following are available online at https://www.mdpi.com/article/10.3390/foods13040603/s1: Table S1. Formulations and initial cooling rates of laminating fats (LF1–LF4) containing olive pomace oil (OPO) and prepared in batches of 1300 g. Table S2. Crystallization and melting peak enthalpies measured by DSC for the different laminating fats (LFs) containing olive pomace oil (OPO) tested at the beginning and end of the chilling storage period in comparison to the ingredients PS and cocoa butter and the controls CB and CLF. Figure S1. Effect of frequency on complex modulus (*G**) for laminating fats after 60 days of chilling storage in comparison with controls CB and CLF, and examples of potential fits to the weak gel model. CB, control commercial butter; CLF, control commercial laminating fat; LF1–LF4, laminating fats formulated with olive pomace oil (OPO). Figure S2. Conical penetration work (spreadability) values at 20 °C for the different laminating fats tested during chilling storage. CB, control commercial butter; CLF, control commercial laminating fat; LF1–LF4, laminating fats formulated with olive pomace oil (OPO). Figure S3. Color values (*L**, *a**, and *b**) for the different laminating fats tested during chilling storage. CB, control commercial butter; CLF, control commercial laminating fat; LF1–LF4, laminating fats formulated with olive pomace oil (OPO). Figure S4. Lipid oxidation (TBARS) for the different laminating fats LF1–LF4 formulated with olive pomace oil (OPO) tested during chilling storage in comparison with control commercial butter (CB) and control commercial laminating fat (CLF).

## References

[1] Silow C., Zannini E., Axel C., Lynch K.M., Arendt E.K. (2016). Effect of Salt Reduction on Wheat-Dough Properties and Quality Characteristics of Puff Pastry with Full and Reduced Fat Content. Food Res. Int..

[2] Wickramarachchi K.S., Sissons M.J., Cauvain S.P. (2015). Puff Pastry and Trends in Fat Reduction: An Update. Int. J. Food Sci. Technol..

[3] Queirós M.S., Grimaldi R., Gigante M.L. (2016). Addition of Olein from Milk Fat Positively Affects the Firmness of Butter. Food Res. Int..

[4] Viriato R.L.S., Queirós M.d.S., Neves M.I.L., Ribeiro A.P.B., Gigante M.L. (2019). Improvement in the Functionality of Spreads Based on Milk Fat by the Addition of Low Melting Triacylglycerols. Food Res. Int..

[5] Litz B., Obert G., Szily B. (2006). Examination of the Correlation of Butter Spreadability and its Fat Conformation by DSC. J. Therm. Anal. Calorim..

[6] Aini I.N., Miskandar M.S. (2007). Utilization of Palm Oil and Palm Products in Shortenings and Margarines. Eur. J. Lipid Sci. Technol..

[7] Detry R., Van Hoed V., Sterckx J., Deledicque C., Sato K., Blecker C., Sabine D. (2021). Physicochemical Properties of Palm Oil-Based Puff Pastry Model Margarines Related to Their Baking Performance in Long-Term Storage. Eur. J. Lipid Sci. Technol..

[8] Nguyen V., Rimaux T., Truong V., Dewettinck K., Van Bockstaele F. (2020). Granular Crystals in Palm Oil Based Shortening/Margarine: A Review. Cryst. Growth Des..

[9] Saadi S., Ariffin A.A., Ghazali H.M., Miskandar M.S., Boo H.C., Abdulkarim S.M. (2012). Application of Differential Scanning Calorimetry (DSC), HPLC and pNMR for Interpretation Primary Crystallisation Caused by Combined Low and High Melting TAGs. Food Chem..

[10] Nguyen V., Rimaux T., Truong V., Dewettinck K., Van Bockstaele F. (2020). Fat Crystallization of Blends of Palm Oil and Anhydrous Milk Fat: A Comparison between Static and Dynamic-Crystallization. Food Res. Int..

[11] Nguyen V., Rimaux T., Truong V., Dewettinck K., Van Bockstaele F. (2021). The Effect of Cooling on Crystallization and Physico-Chemical Properties of Puff Pastry Shortening Made of Palm Oil and Anhydrous Milk Fat Blends. J. Food Eng..

[12] Mateos R., Sarria B., Bravo L. (2020). Nutritional and other health properties of olive pomace oil. Crit. Rev. Food Sci. Nutr..

[13] González-Rámila S., Mateos R., García-Cordero J., Seguido M.A., Bravo-Clemente L., Sarriá B. (2022). Olive Pomace Oil versus High Oleic Sunflower Oil and Sunflower Oil: A Comparative Study in Healthy and Cardiovascular Risk Humans. Foods.

[14] González-Rámila S., Sarriá B., Seguido M.A., García-Cordero J., Bravo L., Mateos R. (2022). Effect of olive pomace oil on cardiovascular health and associated pathologies. Nutrients.

[15] Holgado F., Ruiz-Méndez M.V., Velasco J., Márquez-Ruiz G. (2021). Performance of Olive-Pomace Oils in Discontinuous and Continuous Frying. Comparative Behavior with Sunflower Oils and High-Oleic Sunflower Oils. Foods.

[16] Álvarez M.D., Herranz B., Saiz A., Cofrades S. (2023). Functionality of Puff Pastry Olive Pomace Oil-Based Margarines and their Baking Performance. Foods.

[17] Velasco J., García-González A., Zamora R., Hidalgo F.J., Ruiz-Méndez M.V. (2023). Quality and Nutritional Changes of Traditional Cupcakes in the Processing and Storage as a Result of Sunflower Oil Replacements with Refined Olive Pomace Oil. Foods.

[18] CEE (2002). Commission Regulation (EU) No 1019/2002 of 13 June 2002 on marketing standards for olive oil. Off. J. Eur. Union.

[19] Zamora R., Gómez G., Dobarganes M.C., Hidalgo F.J. (2002). Oil Fractionation as a Preliminary Step in the Characterization of Vegetable Oils by High-Resolution ^13^C NMR Spectroscopy. J. Am. Oil Chem. Soc..

[20] Caponio F., Giarnetti M., Summo C., Gomes T. (2011). Influence of the Different Oils Used in Dough Formulation on the Lipid Fraction of *Taralli*. J. Food Sci..

[21] González-Rámila S., Sarriá B., Seguido M.A., García-Cordero J., Mateos R., Bravo L. (2022). Olive pomace oil can improve blood lipid profile: A randomized, blind, crossover, controlled clinical trial in healthy and at-risk volunteers. Eur. J. Nutr..

[22] Ruiz-Méndez M.-V., Márquez-Ruiz G., Holgado F., Velasco J. (2021). Stability of Bioactive Compounds in Olive-Pomace Oil at Frying Temperature and Incorporation into Fried Foods. Foods.

[23] Álvarez M.D., Cofrades S., Pérez-Mateos M., Saiz A., Herranz B. (2022). Development and Physico-Chemical Characterization of Healthy Puff Pastry Margarines Made from Olive-Pomace Oil. Foods.

[24] International Olive Council (2017). Determination of the Difference between the Actual and Theoretical Content of Triacylglycerols with ECN 42.

[25] Silow C., Zannini E., Axel C., Belz M.C.E., Arendt E.K. (2017). Optimization of Fat-Reduced Puff Pastry Using Response Surface Methodology. Foods.

[26] Silow C., Zannini E., Arendt E.K. (2016). Impact of Low-Trans Fat Compositions on the Quality of Conventional and Fat-Reduced Puff Pastry. J. Food Sci. Technol..

[27] AOAC International (2005). Official Methods of Analysis of AOAC International.

[28] Gabriele D., de Cindio B., D’Antona P. (2001). A Weak Gel Model for Foods. Rheol. Acta.

[29] Genccelep H., Saricaoglu F.T., Anil M., Agar B., Turhan S. (2015). The effect of starch modification and concentration on steady-state and dynamic rheology of meat emulsions. Food Hydrocoll..

[30] Da Pieve S., Calligaris S., Co E., Nicoli M.C., Marangoni A.G. (2010). Shear Nanostructuring of Monoglyceride Organogels. Food Biophys..

[31] Freire M., Cofrades S., Pérez-Jiménez J., Gómez-Estaca J., Jiménez-Colmenero F., Bou R. (2018). Emulsion Gels Containing n-3 Fatty Acids and Condensed Tannins Designed as Functional Fat Replacers. Food Res. Int..

[32] Cofrades S., Hernández-Martín M., Garcimartín A., Saiz A., López-Oliva M.E., Benedí J., Álvarez M.D. (2023). Impact of Silicon Addition on the Development of Gelled Pork Lard Emulsions with Controlled Lipid Digestibility for Application as Fat Replacers. Gels.

[33] Bronzini de Caraffa V., Gambotti C., Giannettini J., Maury J., Berti L. (2008). Using Lipid Profiles and Genotypes for the Characterization of Corsican Olive Oils. Eur. J. Lipid Sci. Technol..

[34] Noor Lida H.M.D., Sundrama K., Siewa W.L., Aminah A., Mamot S. (2002). TAG Composition and Solid Fat Content of Palm Oil, Sunflower Oil, and Palm Kernel Olein Blends Before and After Chemical Interesterification. J. Am. Oil Chem. Soc..

[35] Noor Lida H.M.D., Sundrama K., Nor Aini I. (2007). Effect of Chemical Interesterification on Triacylglycerol and Solid Fat Contents of Palm Stearin, Sunflower Oil and Palm Kernel Olein Blends. Eur. J. Lipid Sci. Technol..

[36] Álvarez M.D., Cofrades S., Espert M., Sanz T., Salvador A. (2021). Development of Chocolates with Improved Lipid Profile by Replacing Cocoa Butter with an Oleogel. Gels.

[37] Kurvinen J.-P., Sjövall O., Tahvonen R., Anklam E., Kallio H. (2002). Rapid MS Method for Analysis of Cocoa Butter TAG. J. Am. Oil Chem. Soc..

[38] Si X., Zhu H., Zhu P., Wang Y., Pang X., Ju N., Lv J., Zhang S. (2023). Triacylglycerol Composition and Thermo-Dynamic Profiles of Fractions from Dry Fractionation of Anhydrous Milk Fat. J. Food Compos. Anal..

[39] Makeria M., Sahric M.M., Ghazalia H.M., Ahmad K., Muhammad K. (2019). Polymorphism, Textural and Crystallization Properties of Winged Bean (*Psophocarpus tetragonolobus, D.C*) Oil-Based Trans-Fatty Acids Free Ternary Margarine Blends. LWT Food Sci. Technol..

[40] Salas Sotaminga Y., Tapia I., Garzón M. (2011). Cristalización y Plastificación de Margarina Industrial para Panificación. Quim. Cent..

[41] Podchong P., Sonwai S., Rousseau D. (2018). Margarines Produced from Rice Bran Oil and Fractionated Palm Stearin and Their Characteristics During Storage. J. Am. Oil Chem. Soc..

[42] Hayati N., Aminah A., Mamot S. (2000). Melting Characteristics and Solid Content of Milk Fat and Palm Stearin Blends Before and After Enzymatic Interesterification. J. Food Lip..

[43] Solo-de-Zaldívar B., Tovar C.A., Borderías A.J., Herranz B. (2015). Pasteurization and Chilled Storage of Restructured Fish Muscle Products Based on Glucomannan Gelation. Food Hydrocoll..

[44] Patel A.R., Nicholson R.A., Marangoni A.G. (2020). Applications of fat mimetics for the replacement of saturated and hydrogenated fat in food products. Curr. Opin. Food Sci..

[45] Mancini F., Montanari L., Peressini D., Fantozzi P. (2002). Influence of Alginate Concentration and Molecular Weight on Functional Properties of Mayonnaise. LWT Food Sci. Technol..

[46] Lupi F.R., Gabriele D., De Cindio B., Sánchez M.C., Gallegos C. (2011). A rheological analysis of structured water-in-olive oil emulsions. J. Food Eng..

[47] Miskandar M.S., Zaliha O., Noor Lida H.M.D., Rafidah ABD H., Sivaruby K., Rosnani O. (2018). Quality Soft Table Margarine with Minimal Post-Crystallization Through High-Pressure Pin-Rotor Unit. J. Oil Palm Res..

[48] Silva T.J., Fernandes G.D., Bernardinelli O.D., da Rosa Silva E.C., Barrera-Arellano D., Ribeiro A.P.B. (2021). Organogels in low-fat and high-fat margarine: A study of physical properties and shelf life. Food Res. Int..

[49] Öğütcü M., Arifoğlu N., Yılmaz E. (2015). Preparation and Characterization of Virgin Olive Oil-Beeswax Oleogel Emulsion Products. J. Am. Oil Chem. Soc..

[50] Çiftҫi O.N., Göğüs F., Fadıloğlu S. (2010). Performance of a Cocoa Butter-Like Fat Enzymatically Produced from Olive Pomace Oil as a Partial Cocoa Butter Replacer. J. Am. Oil Chem. Soc..

[51] Yılmaz E., Öğütcü M. (2014). Properties and Stability of Hazelnut Oil Organogels with Beeswax and Monoglyceride. J. Am. Oil Chem. Soc..

[52] Pirsa S., Asadi S. (2021). Innovative Smart and Biodegradable Packaging for Margarine based on a Nano Composite Polylactic Acid/Lycopene Film. Food Addit. Contam. Part A.

[53] Han Lyn F., Ping Tan C., Zawawi R.M., Nur Hanani Z.A. (2021). Physicochemical Properties of Chitosan/Graphene Oxide Composite Films and their Effects on Storage Stability of Palm-Oil Based Margarine. Food Hydrocoll..

