# Supplementary material for: Olive Pomace Oil Structuring for the Development of Healthy Puff Pastry Laminating Fats: The Effect of Chilling Storage on the Quality of Baked Products
Source: Foods. 2024 Feb 16;13(4):603. doi: 10.3390/foods13040603 (PMC10888308; doi:10.3390/foods13040603)
Supplement: Supplementary file 1 [file foods-13-00603-s001.zip › foods-2811546-supplementary.pdf]

Table S1. Formulation and initial cooling rate of laminating fats (LF1-LF4) containing olive pomace oil (OPO) and prepared in batches of 1300 g.

Formulation	Initial Cooling Rate (°C/min)	Oil Phase, OP (80%)									Aqueous Phase, AP (20%)					
		OPO	Palm Stearin (PS)	Cocoa Butter	Table Butter	Beeswax	Verol P	Verolec Non GMO IP	Palsgaard 1311®	Verol N-90	Water	Salt	Dietary Gelatin	Butter Flavor	Tartaric Acid	Citric Acid
**LF1**	0.144	40.8	23.5	-	7.5	3.0	1.75	1.5	1.2	0.83	17.9	1.0	0.50	0.37	0.25	0.0025
**LF2**	0.380	40.8	23.5	-	7.5	3.0	1.75	1.5	1.2	0.83	17.9	1.0	0.50	0.37	0.25	0.0025
**LF3**	0.144	30.8	23.5	10	7.5	3.0	1.75	1.5	1.2	0.83	17.9	1.0	0.50	0.37	0.25	0.0025
**LF4**	0.380	30.8	23.5	10	7.5	3.0	1.75	1.5	1.2	0.83	17.9	1.0	0.50	0.37	0.25	0.0025

Composition expressed as percentage (%) of the total of ingredients constituting both OP and AP.

Table S2. Crystallization and melting peak enthalpies measured by DSC for the different laminating fats (LF) containing olive pomace oil (OPO) tested at the beginning and end of the chilling storage period in comparison with the ingredients PS and cocoa butter, and controls CB and CLF.

Laminating Fat	Chilling Time (days)	ΔH_{cp1} (J/g)	ΔH_{cp2}(J/g)	ΔH_{mp1} (J/g)	ΔH_{mp2} (J/g)	ΔH_{mp3} (J/g)	ΔH_{mp4} (J/g)
**PS**	-	56.2±0.82	3.91±0.32	3.05±0.51	8.82±0.41	54.7±3.7	-
**Cocoa Butter**	-	-	50.2±3.0	65.0±1.9	-	-	-
**CB**	-	3.30±0.18	10.3±0.78	6.68±0.58	4.14±0.080	3.22±0.50	-
**CLF**	-	17.0±0.38	13.8±0.81	3.31±0.17	3.27±0.21	7.56±1.1	2.27±0.29
**LF1**	3	13.9±0.71 [b]	-	0.372±0.040 [a]	0.422±0.053 [a]	14.4±0.52 [a]	-
**LF2**	3	16.0±0.41 [a]	-	0.285±0.055 [b]	0.462±0.031 [a]	16.3±0.96 [a]	-
**LF3**	3	14.6±0.63 [b]	6.11±0.084 [b]	0.694±0.039 [a]	0.384±0.029 [a]	6.18±0.81 [a]	-
**LF4**	3	16.4±0.79 [a]	6.20±0.24 [a]	0.719±0.056 [a]	0.420±0.029 [a]	6.93±0.12 [a]	-
**LF1**	60	17.8±0.36 [a]	-	0.325±0.032 [a]	0.339±0.035 [a]	15.0±0.24 [a]	-
**LF2**	60	16.5±0.55 [a]	-	0.491±0.031 [a]	0.324±0.0089 [b]	13.3±0.97 [b]	-
**LF3**	60	18.5±0.92 [a]	7.11±0.43 [a]	0.674±0.055 [a]	0.435±0.020 [a]	5.29±0.54 [a]	-
**LF4**	60	18.2±0.81 [a]	6.54±0.29 [a]	0.723±0.019 [a]	0.409±0.016 [a]	4.97±0.22 [b]	-

Mean values (n = 3) ± standard deviation. DSC, differential scanning calorimetry; Olive pomace oil (OPO); PS, palm stearin; CB, control commercial butter; CLF, control commercial laminating fat; LF1-LF4, laminating fats formulated OPO; ΔH_{cp1}, ΔH_{cp2}, enthalpies corresponding to peak temperatures detected from crystallization thermograms; ΔH_{mp1}, ΔH_{mp2}, ΔH_{mp3}, ΔH_{mp4}, enthalpies corresponding to peak temperatures detected from melting thermograms. [a,b] Different letters in the same column and for the same margarine indicate significant differences ($p < 0.05$).

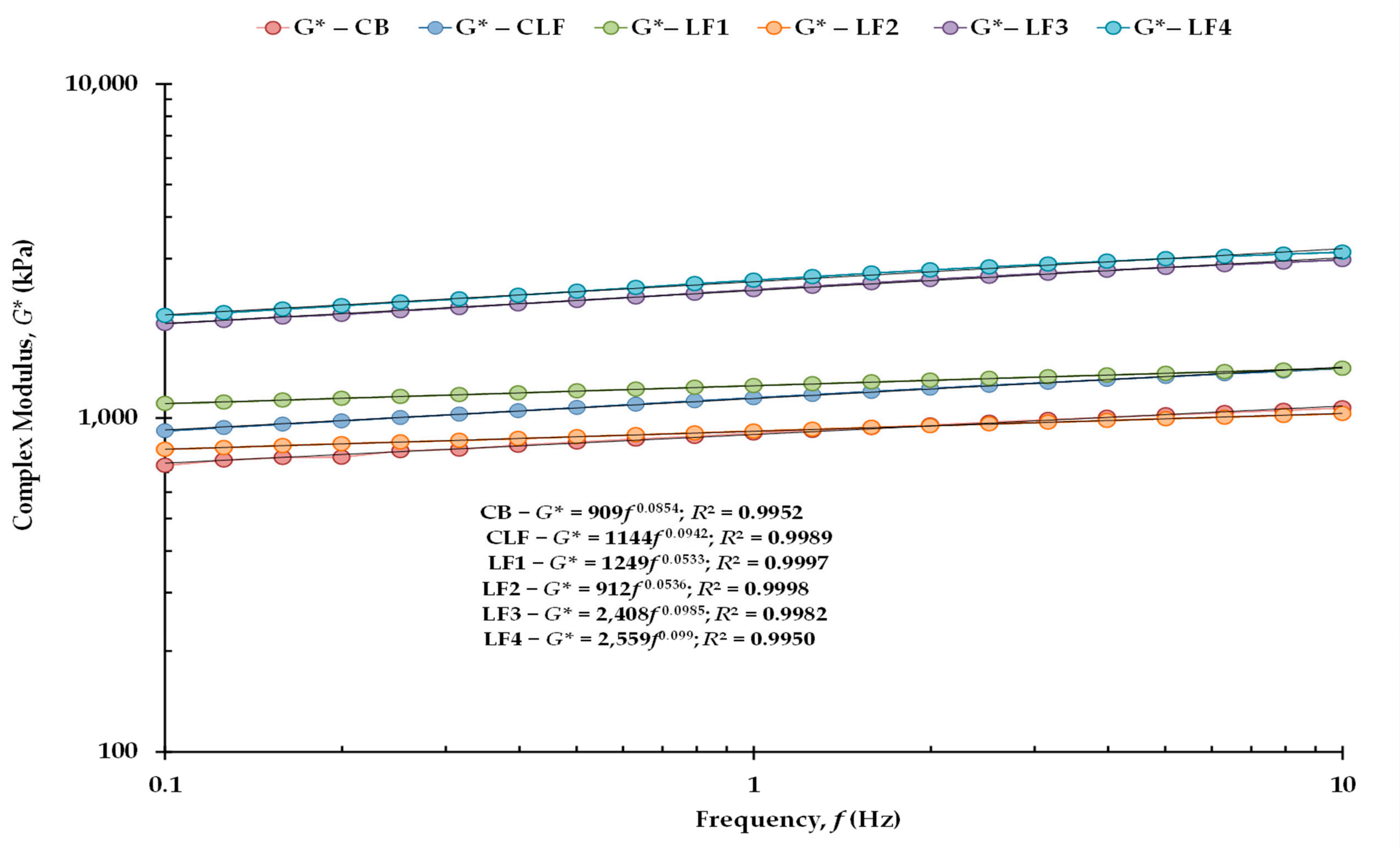

Figure S1. Effect of frequency on the complex modulus (G^*) of laminating fats after 60 days of chilling storage in comparison with controls CB and CLF, and examples of potential fits to the weak gel model. CB, control commercial butter; CLF, control commercial laminating fat; LF1-LF4, laminating fats formulated with olive pomace oil (OPO).

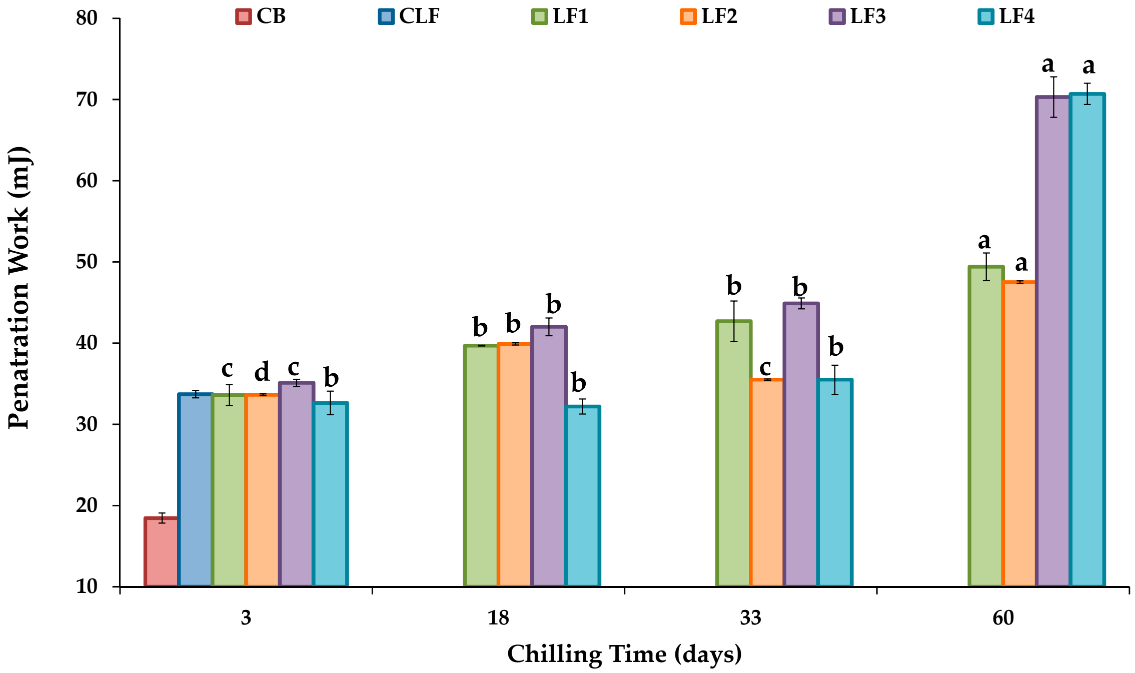

Figure S2. Conical penetration work (spreadability) values at 20 °C for the different laminating fats tested during chilling storage. CB, control commercial butter; CLF, control commercial laminating fat; LF1-LF4, laminating fats formulated with olive pomace oil (OPO). Different letters for the same LF during chilling storage indicate significant differences ($p < 0.05$).

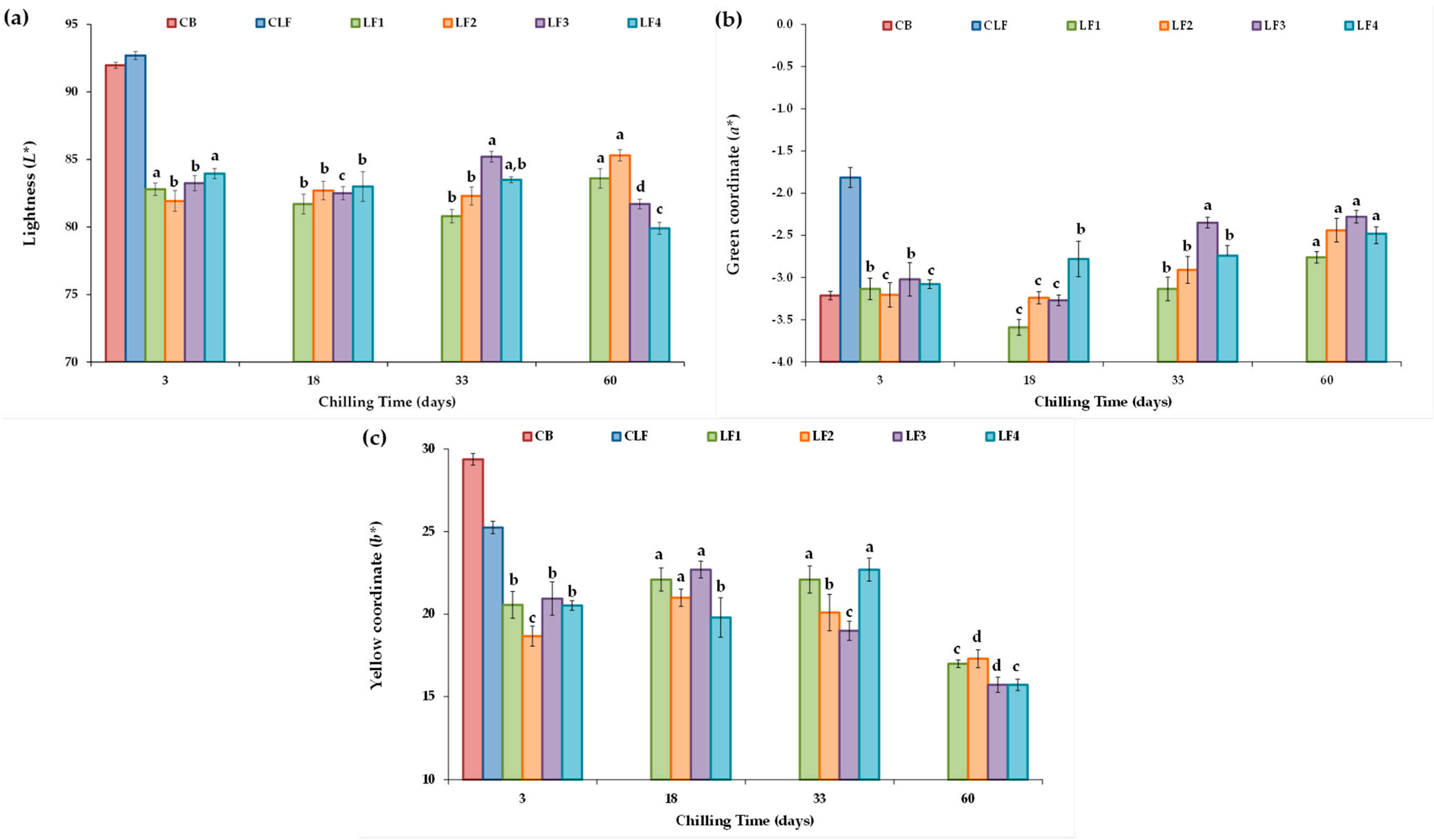

Figure S3. Color values (L^*, a^* and b^*) for the different laminating fats tested along chilling storage. CB, control commercial butter; CLF, control commercial laminating fat; LF1-LF4, laminating fats formulated with olive pomace oil (OPO). Different letters for the same LF during chilling storage indicate significant differences ($p < 0.05$).

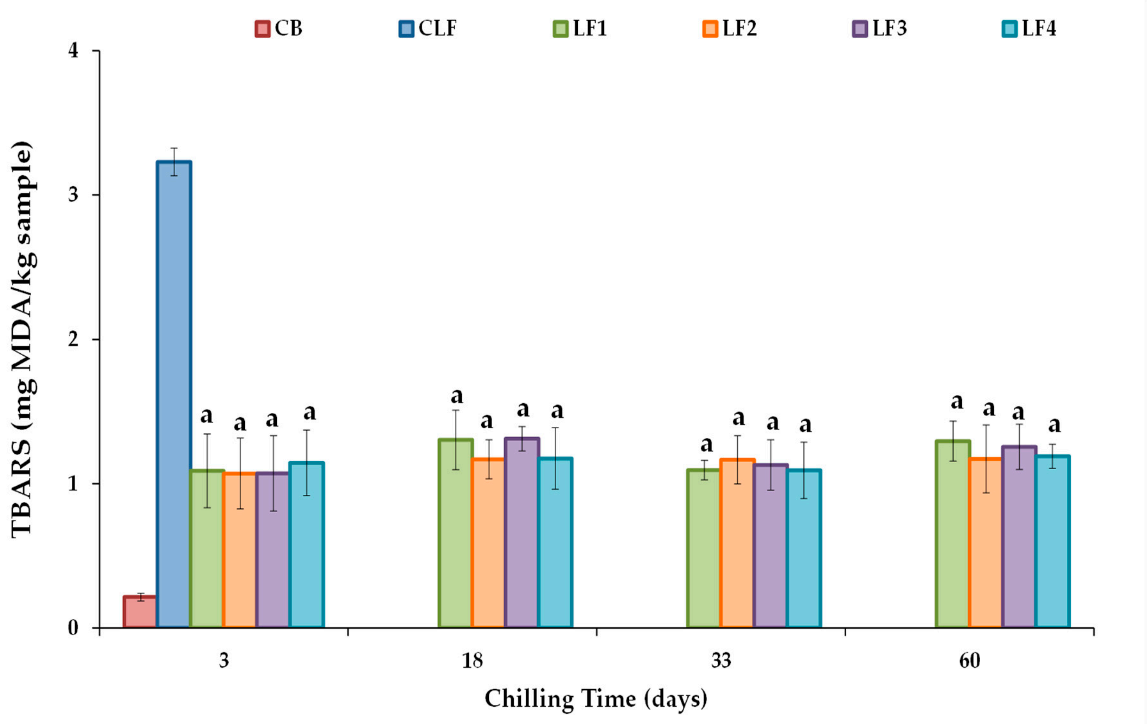

Figure S4. Lipid oxidation (TBARS) for the different laminating fats LF1-LF4 formulated with olive pomace oil (OPO) tested during chilling storage in comparison with control commercial butter (CB) and control commercial laminating fat (CLF). The same letter for each LF during chilling storage indicates no significant differences ($p > 0.05$).
